# Syk Activity Is Dispensable for Platelet GP1b-IX-V Signaling

**DOI:** 10.3390/ijms18061238

**Published:** 2017-06-09

**Authors:** Rachit Badolia, John C. Kostyak, Carol Dangelmaier, Satya P. Kunapuli

**Affiliations:** 1 Department of Physiology, Temple University, Room 414 MRB, 3420 N. Broad Street, Philadelphia, PA 19140, USA; tue39600@temple.edu (R.B.); jck@temple.edu (J.C.K.); cdangelm@temple.edu (C.D.); 2 Sol Sherry Thrombosis Research Center, Temple University, Room 414 MRB, 3420 N. Broad Street, Philadelphia, PA 19140, USA; 3 Department of Pharmacology, Temple University School of Medicine, Philadelphia, PA 19140, USA

**Keywords:** platelets, GP1b receptor, von Willebrand factor (VWF), spleen tyrosine kinase (Syk)

## Abstract

The binding of von Willebrand factor (VWF) to the platelet membrane glycoprotein 1b-IX (GP1b-IX) leads to activation of platelets. GP1b was shown to signal via the FcRγ-ITAM (Fc Receptor γ-Immunoreceptor tyrosine-based activation motif) pathway, activating spleen tyrosine kinase (Syk) and other tyrosine kinases. However, there have been conflicting reports regarding the role of Syk in GP1b signaling. In this study, we sought to resolve these conflicting reports and clarify the role of Syk in VWF-induced platelet activation. The inhibition of Syk with the selective Syk inhibitors, OXSI-2 and PRT-060318, did not inhibit VWF-induced platelet adhesion, agglutination, aggregation, or secretion. In contrast, platelets stimulated with the Glycoprotein VI (GPVI) agonist, collagen-related peptide (CRP), failed to cause any aggregation or secretion in presence of the Syk inhibitors. Furthermore, GP1b-induced platelet signaling was unaffected in the presence of Syk inhibitors, but GPVI-induced signaling was abolished under similar conditions. Thus, we conclude that Syk kinase activity does not play any functional role downstream of GP1b-mediated platelet activation.

The process of platelet activation is an important component of normal hemostasis [1]. The initial adhesion and activation of platelets under high shear conditions of blood flow in the arteries is dependent on their interactions with von Willebrand factor (VWF) [2]. At the site of vascular injury, VWF is a mandatory component of platelet plug formation through its interactions with platelet surface glycoprotein (GP) complex GP1b-V-IX [2,3]. The interaction between VWF and GP1b-IX-V (GP1b) not only mediates transient platelet adhesion but also initiates a signaling cascade leading to platelet integrin α_IIb_β_3_ activation and consequent stable platelet adhesion, spreading, and aggregation [4,5,6]. In vitro, snake venom proteins, ristocetin or botrocetin can modify the interactions between the VWF and GP1b complex to trigger signaling events in human or mouse, respectively. Thus, addition of VWF to the platelets in the presence of ristocetin or botrocetin results in platelet agglutination followed by platelet activation.

A number of signaling pathways have been implicated downstream of GP1b activation upon stimulation of platelets with VWF [7], however, the platelet activation responses are weak when compared with that of other platelet agonists such as thrombin, collagen, and adenosine diphosphate (ADP). GP1b was shown to be constitutively but loosely associated with the Fc receptor γ (FcRγ) chain [8]. Interactions between GP1b and VWF appear first to generate thromboxane A2, which leads to ADP secretion and fibrinogen receptor activation [9]. However, there is a delay in the VWF-GP1b-mediated platelet activation process, which occurs only after near-completion of agglutination. The exact mechanism of GP1b-IX-mediated platelet activation remains unclear, although several intracellular signaling molecules and pathways have been implicated, including the phosphatidyl inositol 3-kinase (PI3-kinase)-protein kinase B (Akt) pathway [10,11,12], the mitogen-activated protein kinase (MAPK) pathways [13,14], and the FcRγ-Syk/PLCγ2 pathway [6,8,15].

It has been reported in multiple studies that Syk is activated downstream of GP1b-VWF interactions [16,17], mostly via GP1b-associated FcRγ-Immunoreceptor tyrosine-based activation motif (ITAM)-mediated signaling [18]. However, another study indicated that the FcRγ chain or FcγRIIa does not play an important role in GP1b signaling, thereby ruling out the role of Syk in GP1b signaling, as Syk requires phosphorylated ITAMs to become activated [19]. On the contrary, a study by Liu J. et al. [20] showed that Syk is required for botrocetin/VWF-induced GP1b signaling by using Syk knockout murine platelets. Subsequent reports using platelets treated with the Syk inhibitor, piceatannol, reported normal adhesion under shear stress, suggesting that stable platelet adhesion to VWF is independent of Syk [21].

In this study, we evaluated the role of Syk in VWF signaling in human platelets by using two different small molecule pharmacological inhibitors of Syk, PRT 060318 (or PRT-318) (2-((1*R*,2*S*)-2-aminocyclohexylamino)-4-(m-tolylamino) pyrimidine-5-carboxamide) and OXSI-2 (2,3-dihydro-3-[(1-methyl-1*H*-indol-3-yl) methylene]-2-oxo-1*H*-indole-5-sulfonamide). Both the inhibitors are adenosine triphosphate (ATP)-competitive inhibitors and inhibit the kinase-activity of Syk [22]. As shown in Figure 1A, VWF, in the presence of ristocetin, induced platelet agglutination followed by a second wave of aggregation and secretion, mediated by generated thromboxane A2 (TxA2). However, washed human platelets pretreated with either of the Syk inhibitors, OXSI-2 or PRT-060318 (PRT-318), resulted in normal agglutination, aggregation, and secretion comparable to the vehicle control, DMSO (Dimethyl sulfoxide). As shown in Figure 1B, under the same experimental conditions, both OXSI-2 and PRT-060318 abolished the GPVI agonist, as well as collagen-related peptide (CRP)-induced platelet aggregation and secretion. This confirms that Syk inhibitors are effective and, unlike the GPVI pathway where Syk has a crucial proximal role, GP1b-induced platelet agglutination, aggregation, and secretion is unaffected. Additionally, if the Syk inhibitors used in our study had any non-specific inhibitory effects on Src family kinases, we would not have observed any aggregation or secretion with ristocetin/VWF, as Src family kinases are essential for GP1b-mediated platelet activation [13,18].

This result contradicts the earlier report by Liu J et al. [20], where it was shown that *Syk*^−/−^ murine platelets agglutinated normally with VWF/botrocetin, but failed to aggregate or secrete ATP. Our results suggest that the initial GP1b signaling does not depend on Syk kinase activity. It is possible that in Syk-deficient mice, the expression of upstream molecules or regulators of VWF signaling at the level of progenitor cells of megakaryocytic lineage might be affected, resulting in the observed phenotype. In the same study, it was also reported that agglutination-elicited TxA2 production does not require FcRγ [20]. This further supports our finding that Syk is not involved in the GP1b signaling, as Syk is activated via the FcRγ-pathway upon GP1b stimulation [18]. Since both the Syk inhibitors inhibit only the kinase activity of Syk, but not the docking of Syk to the phosphorylated tyrosine residues of the receptor, it could be possible that Syk kinase activity might not be required for GP1b-mediated signaling. However, in a likely possibility, the Syk bound to the receptor complex might have other functions such as scaffolding or adaptor functions. Hence, the complete absence of Syk in *Syk*^−^/^−^ mice results in the complete inhibition of GP1b-mediated platelet aggregation and secretion [20]. However, there was no effect on GP1b-mediated adhesion of human platelets treated with the Syk inhibitor [21].

To determine the effect of Syk inhibition on GP1b signaling, Western blot analysis was performed on the platelet lysates obtained after stimulation with either VWF/ristocetin or CRP (as a control) in the presence or absence of the Syk inhibitors. Figure 1C shows that Syk causes the phosphorylation of tyrosine at Y352 residue by VWF/ristocetin and is not affected by the inhibition of Syk kinase activity. This agrees with the previous reports, which identified Syk Y352 as a site phosphorylated by Src family kinases (SFK) (which lies upstream of Syk) upon receptor activation [23]. Furthermore, the intact phosphorylation of Syk Y352 in the presence of the Syk inhibitors suggests that the inhibitors are selective for Syk and do not have any non-specific effect on the upstream SFKs. However, the downstream signaling events, including the phosphorylation of PLCγ2, are only slightly inhibited, whereas Akt and Erk1/2 phosphorylation are unaffected in the presence of both OXSI-2 and PRT-318. Under the same experimental conditions, CRP-induced signaling downstream of Syk is abolished in the presence of the Syk inhibitors, but not in the presence of SFK-mediated Syk Y352 phosphorylation (Figure 1D). These results suggest that, although Syk kinase activity has a crucial role in GPVI signaling, it does not play any functional role in GP1b-IX-V signaling.

Additionally, to identify the role of Syk kinase activity in the initial adhesion of platelets to the immobilized VWF, we performed a flow over VWF assay. As shown in Figure 2, there is no significant difference in the number of adhered platelets to immobilized VWF in the presence of the Syk inhibitors, OXSI-2 (1 µM) and PRT-318 (1 µM), as compared to the DMSO control. This result supports our aggregation/secretion data, where the agglutination of platelets was not affected in the presence of Syk inhibitors, and suggests that Syk activity does not play a role in the initial adhesion of platelets to VWF. Interestingly, the initial agglutination was normal even in *Syk*^−^/^−^ murine platelets [20], further supporting our result.

Syk-independent pathways in platelet activation downstream of GP1b have also been suggested in previous reports. Yin et al. [21] showed that the ITAM-Syk pathway does not play a predominant role in Tyrosine-protein kinase Lyn-dependent GP1b signaling, leading to integrin activation. Although PLCγ2 is known to be the downstream substrate of Syk, we showed in this study that PLCγ2 phosphorylation is only partially affected in the presence of Syk inhibitors, suggesting a Syk-independent pathway of PLCγ2 activation. Previously, it was shown that PLCγ2 could be activated by a PI3-Kinase-dependent mechanism without the requirement for Syk [20]. VWF-induced PI3-Kinase activation is required for most of the phosphorylation of PLCγ2 [20], and the direct activation of PLCγ2 by PI3-Kinase has also been reported [24,25,26,27]. Similarly, it has also been suggested that Erk and Akt were activated in a FcRγ- or Syk-independent manner by PI3-kinase, downstream of Lyn upon VWF-GP1b interaction [21].

In conclusion, our results demonstrate that Syk, although phosphorylated upon GP1b stimulation, does not play any kinase-dependent functional role in ristocetin/VWF-induced platelet adhesion, aggregation, or secretion, or in GP1b signaling. Thus, not only is GP1b a central player in hemostasis, but it may also be able to signal by an essentially different mechanism than other ITAM-pathways in platelets. The results presented in this study extend our knowledge of the VWF-GP1b signaling pathways in platelets and support the evidence that Syk kinase activity is not required for GP1b signaling. Understanding the versatility of GP1b signaling may therefore prove helpful in developing a rationale for the design of clinically useful antithrombotic agents targeted to the GP1b function.

## Figures and Tables

**Figure 1:**
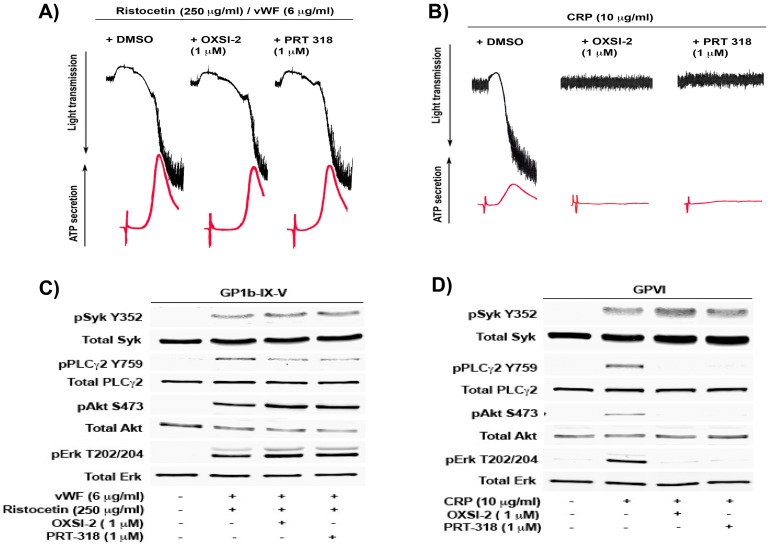
Inhibition of Syk does not inhibit GP1b-mediated platelet aggregation, secretion, and signaling: Washed non-aspirin human platelets were pre-incubated for 5 min with either dimethyl sulfoxide (DMSO) as control, 2,3-dihydro-3-[(1-methyl-1*H*-indol-3-yl) methylene]-2-oxo-1*H*-indole-5-sulfonamide (OXSI-2) (1 µM), or 2-((1*R*,2*S*)-2-aminocyclohexylamino)-4-(m-tolylamino) pyrimidine-5-carboxamide (PRT-318) (1 µM) and stimulated with (**A**) ristocetin/von Willebrand factor (VWF) (250 and 6 µg/mL respectively); (**B**) Collagen related peptide (CRP) (10 µg/mL), for 5 min at 37 °C under stirred conditions in a lumi-aggregometer. The tracings are representative of data from three individual experiments. (The black tracings represent the platelet aggregation while the red lines represent the adenosine triphosphate (ATP) secretion). Washed non-aspirin human platelets were pre-incubated for 5 min with either DMSO (control), OXSI-2 (1 µM) or PRT-060318 (1 µM) and stimulated with (**C**) ristocetin/VWF (250 and 6 µg/mL respectively) for 4 min; (**D**) CRP (10 µg/mL), for 1 min at 37 °C under stirred conditions in a lumi-aggregometer. The reaction was stopped by using 6.6 N Perchloric acid and platelet lysates were prepared. Platelet proteins were separated by SDS-PAGE (Sodium dodecyl sulfate-polyacrylamide gel electrophoresis), transferred on a membrane by Western-blotting, and probed for phospho Syk (Tyr352), PLCγ2 (Tyr759), Akt (Ser473), Erk1/2 (T202/Y204), and respective total proteins as lane loading controls. The Western blot shown is a representative of three independent experiments (Badolia et al.).

**Figure 2:**
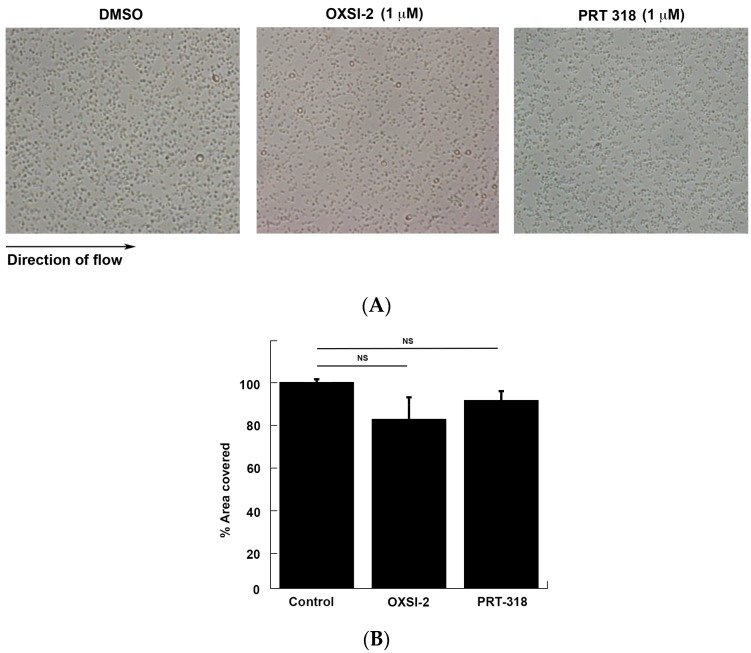
Effect of Syk inhibitors on platelet adhesion to VWF. (**A**) Whole human blood was pretreated with DMSO (control) or OXSI-2 (1 µM) or PRT-318 (1 µM) for 5 min and perfused over immobilized VWF in a flow chamber at an arterial shear rate of 1000 s^−1^. After washing, stably adherent platelets were observed at 20× magnification and photographed using a confocal microscope; (**B**) Quantification of the percentage of area covered by using Image J software (Image Processing and Analysis in Java from National Institute of Health, https://imagej.nih.gov/ij/). Shown in the figure are representative pictures from three different experiments.
